# Cognitive impairments in patients with persistent symptoms attributed to Lyme disease

**DOI:** 10.1186/s12879-019-4452-y

**Published:** 2019-10-07

**Authors:** Anneleen Berende, Joost Agelink van Rentergem, Andrea W. M. Evers, Hadewych J. M. ter Hofstede, Fidel J. Vos, Bart Jan Kullberg, Roy P. C. Kessels

**Affiliations:** 1 Department of Internal Medicine 463 and Radboud Center for Infectious Diseases, Radboud University Medical Center, P.O. Box 9101, 6500 HB Nijmegen, the Netherlands; 2 Department of Psychology, University of Amsterdam, Amsterdam, the Netherlands; 3 Department of Medical Psychology, Radboud University Medical Center, Nijmegen, the Netherlands; 4 Institute of Psychology, Health, Medical, and Neuropsychology Unit, Leiden University, Leiden, the Netherlands; 5 Department of Medicine, Sint Maartenskliniek, Nijmegen, the Netherlands; 6 Donders Institute for Brain, Cognition and Behaviour, Radboud University, Nijmegen, the Netherlands

**Keywords:** Lyme disease, Cognitive neuropsychology, Cognition, Borrelia

## Abstract

**Background:**

Persistent symptoms attributed to Lyme borreliosis often include self-reported cognitive impairment. However, it remains unclear whether these symptoms can be substantiated by objective cognitive testing.

**Methods:**

For this observational study, cognitive performance was assessed in 280 adults with persistent symptoms attributed to Lyme borreliosis (as part of baseline data collected for the Dutch PLEASE study). Cognitive testing covered the five major domains: episodic memory, working memory / attention, verbal fluency, information-processing speed and executive function. Patients’ profiles of test scores were compared to a large age-, education- and sex-adjusted normative sample using multivariate normative comparison. Performance validity was assessed to detect suboptimal effort, and questionnaires were administered to measure self-reported cognitive complaints, fatigue, anxiety, depressive symptoms and several other psychological factors.

**Results:**

Of 280 patients, one was excluded as the test battery could not be completed. Of the remaining 279 patients, 239 (85.4%) displayed sufficient performance validity. Patients with insufficient performance validity felt significantly more helpless and physically fatigued, and less orientated. Furthermore, they had a lower education level and less often paid work. Of the total study cohort 5.7% (*n* = 16) performed in the impaired range. Among the 239 patients who displayed sufficient performance validity, 2.9% (*n* = 7) were classified as cognitively impaired. No association between subjective cognitive symptoms and objective impairment was found.

**Conclusions:**

Only a small percentage of patients with borreliosis-attributed persistent symptoms have objective cognitive impairment. Performance validity should be taken into account in neuropsychological examinations of these patients. Self-report questionnaires are insufficiently valid to diagnose cognitive impairment.

**Trial registration:**

ClinicalTrials.gov NCT01207739. Registered 23 September 2010.

## Background

Patients with persistent symptoms attributed to Lyme borreliosis often report a variety of cognitive symptoms. However, subjective cognitive symptoms are not always due to underlying cognitive impairments. Previously, subjective ratings of memory capabilities and objective memory performance were only weakly correlated in patients with post-treatment Lyme disease [1]. Most previous studies that compared cognitive performance of Lyme patients to performance of healthy controls found a worse performance in Lyme patients at group level [2–12]. The most affected cognitive domain was episodic memory. Findings concerning the domains verbal fluency and processing speed were less consistent. However, most studies were relatively small (*n* < 80), originated from the US, and used diverse inclusion criteria and methods. In the 4 larger US studies, mild to no cognitive abnormalities were identified [1, 2, 12, 13]. As both the *Borrelia* species and the clinical presentation of Lyme disease in Europe and the US differ [14], cognitive function in European Lyme patients requires separate assessment. Furthermore, most previous studies have not taken performance validity into account. This is crucial, as a suboptimal performance results in poor tests scores not reflecting an individual’s actual cognitive status. Very recently, the study by Touradji et al. in a group of US patients with post-treatment Lyme disease showed that 24% of the sample displayed suboptimal effort on measures of performance validity [12]. Hence, suboptimal performance affects the validity and reliability of neuropsychological outcomes, resulting in false positive results (i.e., patients incorrectly labelled as having a cognitive impairment) [15]. This stresses the need to take performance validity testing into account when cognitively assessing patients with persistent symptoms attributed to Lyme disease.

The aim of the present study was to objectively assess cognitive performance using sensitive tests in a large cohort of patients with persistent symptoms attributed to Lyme borreliosis, while taking performance validity into account, and to compare cognitive performance outcomes with subjective symptoms.

## Methods

The current study uses baseline data collected between 2010 and 2013 as part of the Persistent Lyme Empiric Antibiotic Study Europe (PLEASE). Previously, we reported the primary and secondary outcome measures of this multicenter, placebo-controlled, double-blind randomized controlled trial from the Netherlands (ClinicalTrials.gov NCT01207739) [16, 17]. The local Institutional Review Board approved the PLEASE protocol, and informed consent was obtained from each participant. Here we provide a detailed report of the baseline cognitive and self-report questionnaire data. The study population comprises adult patients (*n* = 280) referred with persistent symptoms attributed to Lyme borreliosis, preceded by confirmed symptomatic Lyme disease or accompanied by positive *B. burgdorferi* IgG or IgM antibodies, as confirmed by means of immunoblot assay. Patients were not required to have received antibiotic treatment before study entry. Major symptoms included musculoskeletal pain, cognitive disturbances and/or fatigue. Details about inclusion and exclusion criteria have been published previously [16].

### Outcomes

Cognitive performance was assessed using an extensive neuropsychological test battery covering five major cognitive domains: episodic memory, working memory / attention, verbal fluency, information-processing speed and executive function. Episodic memory was assessed using the Rey Auditory Verbal Learning Test (RAVLT), working memory / attention with the Digit Span, verbal fluency with the Category Fluency Test (animal/profession naming), and information-processing speed with the Trail Making Test Part A and the mean response time of cards I and II from the Stroop Color-Word Test, and the Symbol-Digit Substitution Test. Executive function was measured using the Interference Score of the Trail Making Test (Part B/Part A) and the Stroop Interference Score (card III/mean of cards I and II). Assessment details have been published previously [17].

To identify participants with insufficient performance validity, the Amsterdam Short Term Memory test (ASTM) was administered [18]. A poor performance on this task indicates suboptimal mental effort. The recommended cut-off score is 85 (maximum score = 90), with 86% sensitivity and 87% specificity [19]. However, since our goal was to prioritize optimal specificity (> 90%), adopting a conservative approach that reduces the risk of false alarms on performance validity tests (i.e., incorrectly labelling a participant as someone displaying suboptimal effort), we used a cut-off score of < 83 (which has a specificity of 95% and a sensitivity of 76%).

Subjective measurement of cognitive function was assessed with the Cognitive Failures Questionnaire (CFQ) [20], fatigue by the Checklist Individual Strength (CIS) [16], anxiety and depressive symptoms by the Hospital Anxiety and Depression Scale (HADS) [21], self-efficacy by a modified version of the Arthritis Self-Efficacy Scale (i.e., ‘pain’ replaced by ‘physical symptoms’) [22], illness cognitions by the Illness Cognition Questionnaire (ICQ) [23], worrying by the Penn-State Worry Questionnaire (PSWQ) [24], neuroticism and extraversion by the Eysenck Personality Questionnaire (EPQ) [25], and fear of body sensations by the Body Sensations Questionnaire (BSQ) [26].

### Statistical analysis

First, we investigated which demographic/psychological factors were associated with poor performance validity. For patients with sufficient performance validity, we determined whether their cognitive performance was impaired by comparing individual test performances to an extensive normative sample (*n* = 26,939) from the Advanced Neuropsychological Diagnostics Infrastructure (ANDI) [27]. We performed a multivariate normative comparison (MNC) on each patient’s neuropsychological test profile, applying corrections for age, sex and education level. The MNC provides an individual classification based on the profile of tests as either ‘cognitively impaired’ or ‘cognitively unimpaired’ [27].

We explored performance on individual cognitive domains, averaging age-, sex- and education-adjusted z-scores per domain. A domain was classified as ‘impaired’ if z < − 1.5 (i.e., more than 1.5 SD below the normative mean). The relation between objective cognitive functioning and subjective complaints was analyzed with Pearson correlation coefficients.

Alpha was set at 0.05 throughout (two-tailed), and 95% confidence intervals are reported when appropriate. Benjamini-Hochberg correction was used to reduce the false discovery rate for multiple comparisons, accepting a false discovery rate of 0.10.

## Results

Of the 280 patients included, one was unable to perform several neuropsychological tests due to visual impairment unrelated to Lyme disease. Of the 279 patients fully examined, 239 (85.4%) displayed sufficient performance validity.

Table 1 shows patient characteristics stratified by performance validity status.

**Table 1:** Demographic and psychosocial factors stratified by performance validity^a^

Characteristic	Good performance validity (*n* = 239)	Poor performance validity (*n* = 40)	*P* Value
Women, no. (%)	109 (45.6)	19 (47.5)	0.82
Education level, no. (%)			
Low (≤8 years)	1 (0.4)	0 (0)	0.03
Average (9–11 years)	125 (52.7)	28 (71.8)	
High (≥12 years)	111 (46.8)	11 (28.2)	
Paid work, no. (%)	154 (64.7)	17 (43.6)	0.01
Age, mean (± SD), years	48.7 (11.9)	49.0 (11.9)	0.87
Duration of symptoms, median (IQR), years	2.7 (1.3–6.3)	1.8 (0.7–5.7)	0.13
Previous antibiotic treatment for Lyme disease, no. (%)	213 (89,1)	33 (82.5)	0.23
Delay symptom onset and treatment, median (IQR), weeks	22.5 (3.0–103.5)	15.5 (2.0–69.0)	0.41
History of meningoradiculitis (neuroborreliosis), no. (%)^c^	18 (7.5)	3 (7.7)	0.97
CFQ, mean (95% CI)			
Orientation	3.92 (3.65–4.18)	4.98 (4.20–5.75)	0.004^b^
Distractibility	11.29 (10.71–11.87)	12.98 (11.58–14.37)	0.03
Blunders	7.04 (6.70–7.38)	7.95 (7.15–8.75)	0.05
Memory	7.32 (7.05–7.59)	7.03 (6.28–7.77)	0.43
Total	43.23 (41.52–44.94)	47.73 (43.24–52.21)	0.05
HADS, mean (95% CI)			
Anxiety	6.36 (5.82–6.90)	6.08 (4.90–7.26)	0.71
Depression	7.47 (6.98–7.96)	8.76 (7.33–10.19)	0.06
CIS, mean (95% CI)			
Fatigue severity	43.62 (42.33–44.91)	47.25 (44.38–50.11)	0.03
Concentration	23.84 (22.92–24.76)	25.35 (22.74–27.96)	0.23
Motivation	16.67 (15.92–17.42)	17.93 (15.93–19.92)	0.22
Activity	13.92 (13.27–14.57)	16.15 (14.73–17.57)	0.01^b^
Total	98.03 (95.34–100.72)	106.65 (100.82–112.49)	0.02
Self-efficacy, mean (95% CI)	17.28 (16.60–17.96)	15.62 (13.92–17.31)	0.07
ICQ, mean (95% CI)			
Helplessness	13.26 (12.71–13.80)	15.54 (14.20–16.87)	0.002^b^
Acceptance	13.68 (13.17–14.20)	13.42 (12.27–14.58)	0.70
Perceived benefits	11.35 (10.82–11.88)	12.63 (11.43–13.82)	0.07
EPQ, mean (95% CI)			
Neuroticism	8.27 (7.59–8.96)	7.67 (6.22–9.12)	0.50
Extraversion	11.44 (10.83–12.04)	12.21 (10.60–13.81)	0.34
PSWQ, mean (95% CI)	42.17 (40.55–43.78)	42.15 (38.30–46.00)	0.99
BSQ, mean (95% CI)	2.27 (2.19–2.35)	2.38 (2.17–2.59)	0.33

*Abbreviations*: *BSQ* body sensations questionnaire, *CFQ* Dutch version of the Cognitive Failures Questionnaire, *CIS* checklist individual strength, *EPQ* Eysenck Personality Questionnaire, *HADS* hospital anxiety and depression scale, *ICQ* illness cognition questionnaire, *PSWQ* Penn-State Worry Questionnaire

^a^Between-group differences in characteristics were analyzed with analysis of variance for continuous variables, chi-square tests for proportions, and Kruskal-Wallis tests for ordinal variables and data that were not normally distributed

^b^Significant with the Benjamini-Hochberg correction, accepting a false discovery rate of 0.10

^c^Diagnosis by intrathecal *Borrelia* antibody production

Patients with insufficient performance validity had significantly lower education levels and less often paid work. They also reported significantly more feelings of helplessness (ICQ subscale helplessness, F(1,275) = 9.77), experienced more physical fatigue (CIS Activity subscale, F(1,276) = 6.79), and reported more problems in daily orientation (CFQ Orientation subscale, F(1,276) = 8.40).

Compared to the normative sample, 2.9% of patients (7/239) were cognitively impaired. For the separate domains, 2.1% were impaired on episodic memory (5/239), 5.4% on working memory / attention (13/239), 0.8% on verbal fluency (2/239), 2.1% on information-processing speed (5/239), and 0.4% on executive function (1/239). Table 2 shows the raw neuropsychological test scores as well as mean z-scores, and percentage of individuals with a cognitive decrement (z-score < 1.0 SD below the age, sex and education-adjusted normative mean).

**Table 2:** Neuropsychological test scores per domain and per test^a^

	Good performance validity (*n* = 239)			Poor performance validity (*n* = 40)		
	mean raw score (SD)	mean z-score (SD)	cognitive decrement no. (%)	mean raw score (SD)	mean z-score (SD)	cognitive decrement no. (%)
Episodic memory	− 0.12 (0.71)			− 0.40 (0.81)		
RAVLT (immediate recall, total of trials 1–5)	45.3 (8.4)	−0.12 (0.71)	29 (12.1)	41.0 (8.5)	−0.43 (0.75)	8 (20.0)
RAVLT (delayed recall)	9.4 (2.8)	−0.11 (0.80)	30 (12.6)	8.4 (3.3)	−0.37 (1.01)	9 (22.5)
Working memory / attention	0.11 (1.01)			−0.40 (0.86)		
Digit Span	15.4 (3.3)	0.11 (1.01)	31 (13.0)	13.5 (2.7)	−0.40 (0.86)	10 (25.0)
Language	0.02 (0.74)			−0.04 (0.84)		
Category Fluency (animals)	25.4 (5.8)	−0.03 (0.79)	25 (10.5)	24.3 (6.5)	−0.11 (0.96)	8 (20.0)
Category Fluency (professions)	19.1 (4.8)	0.07 (0.91)	28 (11.7)	18.2 (4.3)	0.03 (0.94)	6 (15.0)
Information-processing speed	0.02 (0.72)			−0.49 (0.93)		
Trail Making Test part A^b^	30.5 (11.5)	0.19 (0.84)	24 (10.0)	33.9 (12.0)	−0.01 (0.85)	6 (15.4)
Stroop Color-Word Test (Card I)^b^	44.1 (8.7)	−0.10 (0.99)	46 (19.3)	48.0 (9.9)	−0.50 (1.13)	8 (21.1)
Stroop Color-Word Test (Card II)^b^	58.9 (12.5)	−0.23 (1.10)	53 (22.3)	66.1 (13.9)	−0.84 (1.18)	16 (42.1)
Symbol-Digit Substitution Test	57.6 (11.3)	0.05 (0.99)	31 (13.0)	49.5 (10.6)	−0.59 (1.10)	11 (27.5)
Executive functions	0.33 (0.68)			0.11 (0.73)		
Trail Making Test interference score (Part B/Part A)^b^	2.3 (0.7)	−0.13 (0.99)	45 (18.8)	2.5 (0.6)	−0.38 (0.92)	9 (23.1)
Stroop interference score (Card III/average Card I and II)^b^	1.8 (0.3)	0.78 (0.71)	4 (1.7)	1.9 (0.3)	0.61 (0.86)	1 (2.6)

*Abbreviations*: *RAVLT* Rey auditory verbal learning test

^a^mean standardized age, sex and education-adjusted normative z-scores (SD) are presented for the domains, mean raw scores (SD), as well as mean z-scores (SD), and percentage of subjects with a cognitive decrement (z-score < 1.0 SD below the age, sex and education-adjusted normative mean) are presented for the separate tests. Higher scores represent better cognitive performance, unless otherwise indicated

^b^higher scores represent worse cognitive performance

No significant correlation between overall cognitive performance and subjective cognitive complaints (CFQ total score) was found in patients with sufficient performance validity (*r* = 0.120, *p* = 0.064). A significant correlation was found between objective performance and problems in orientation (CFQ subscale orientation; *r* = 0.246 (*p* < 0.001). Performance and other CFQ subscales did not show any correlations (*p* = 0.10–0.98).

## Discussion

We have assessed neurocognitive function in the largest sample of patients with persistent symptoms attributed to Lyme disease so far, using sensitive tests and extensive normative data. Furthermore, this study was the first European study to take performance validity into account. In our study, 15% of patients displayed insufficient performance validity using a conservative cut-off that prioritizes specificity over sensitivity (i.e., reducing the chance of incorrectly labelling an individuals as displaying poor performance validity), indicating that their neuropsychological test scores cannot be interpreted reliably as they might not reflect the patients’ actual cognitive abilities. Of note, there remains a possibility that this group with insufficient performance validity contains legitimate poor performers. For example, they may have been too fatigued to perform sufficiently. Our percentage of insufficient performance is considerably lower than in the study of Touradji et al. [12] who found that 24% of their sample of post-treatment Lyme disease patients showed suboptimal effort, which may have been due to our conservative approach in assessing suboptimal effort. These substantial proportions of patients displaying suboptimal effort, however, illustrate the need for performance validity testing when assessing cognition in patients with persistent neuropsychological symptoms [15]. Patients who displayed insufficient effort reported more fatigue, memory-orientation difficulties, and more feelings of helplessness than the optimal performers. Additionally, these patients were more often without paid work and had lower education levels. It should also be noted that only 22.5% (9/40) of the individuals who displayed suboptimal effort would have been classified as ‘cognitively impaired’ based on the performance on all other neuropsychological tests. After exclusion of patients with poor performance validity, only 2.9% had impaired cognitive function. This rate is low compared to the high level of cognitive complaints reported by patients (on average about 1 SD above the normative mean on the CFQ) [20]. The lack of correlation between objective and subjective cognitive functioning, was also reported in another large study in patients with Lyme-associated symptoms [1]. This lack of correlation between objective cognitive performance and subjective cognitive complaints is not specific for Lyme, but has been demonstrated in other disorders as well (including HIV, dementia, and rheumatoid arthritis) [28–30]. Subjective cognitive complaints are often associated with depressive symptoms [29]. The percentage of 2.9% cognitively impaired patients is comparable to what is found in the normal population (i.e., by definition 2.3% of a normative sample performs worse than 2 SD below the normative mean). Similar to our results, the study by Kaplan et al. [1] found only a small percentage of cognitively impaired individuals. That study also examined personality characteristics, albeit with a smaller sample size and a less extensive test battery than the present study. Another large study did not find any differences between patients and healthy controls in cognitive function either [2]. However, a very recent large study, which did take performance validity into account, found a much higher percentage [12]. Furthermore, two studies by Keilp et al. found distinctive cognitive difference between patients with symptoms attributed to Lyme disease and healthy controls [6, 7]. We can only speculate on an explanation for the difference in impairment for the patients with sufficient performance. Possibly, differences between *Borrelia* species in the US and Europe may play a role. In addition, differences in recruitment bias across the various studies may also have played a role. For instance, in the paper by Touradji et al. [12] it is stated that participants partially were self-referred, whereas our patients were all referred to the study centers by a primary care physician or medical specialty.

In addition to the low prevalence of cognitive impairments in our study, the pathogenesis of impaired cognition in relation to Lyme disease is still unclear, with scarce evidence for underlying central nervous system pathology [31].

A potential limitation of the present study is the absence of a contemporaneous control group of healthy individuals. However, we compared the individuals’ performances to a substantially larger normative sample, with specific adjustments for age, education and sex, than would have been ever possible with recruiting our own controls. Additionally, the fact that our study population was more heterogenous than previous studies could be seen as a limitation, and not all patients received previous treatment with antibiotics.

## Conclusions

The present study, taking performance validity into account in a large, well-defined cohort of patients with persistent symptoms attributed to Lyme borreliosis, demonstrates that only a small percentage of patients can be classified as cognitively impaired. Furthermore, self-reported symptoms of cognitive problems are unrelated to performance on neuropsychological tests in patients with Lyme-associated symptoms.

## Data Availability

The datasets used and analysed during the current study are available from the corresponding author on reasonable request.

## Abbreviations

ASTM: Amsterdam short term memory test
BSQ: Body sensations questionnaire
CFQ: Cognitive failures questionnaire
CIS: Checklist individual strength
EPQ: Eysenck personality questionnaire
HADS: Hospital anxiety and depression scale
ICQ: Illness cognition questionnaire
PLEASE: Persistent Lyme Empiric Antibiotic Study Europe
PSWQ: Penn-State Worry Questionnaire
RAVLT: Rey auditory verbal learning test
